# Priority and age specific vaccination algorithm for the pandemic diseases: a comprehensive parametric prediction model

**DOI:** 10.1186/s12911-021-01720-6

**Published:** 2022-01-06

**Authors:** Onder Tutsoy, Mahmud Yusuf Tanrikulu

**Affiliations:** 1 Department of Electreical-Electronics Engineering, Adana Alparslan Turkes Science and Technology University, Adana, 01250 Turkey; 2 METU MEMS Center, Middle East Technical University, Ankara, 06800 Turkey

**Keywords:** COVID-19, Pandemic, Priority and age specific vaccination policy, Parametric model, Prediction

## Abstract

**Background:**

There have been several destructive pandemic diseases in the human history. Since these pandemic diseases spread through human-to-human infection, a number of non-pharmacological policies has been enforced until an effective vaccine has been developed. In addition, even though a vaccine has been developed, due to the challenges in the production and distribution of the vaccine, the authorities have to optimize the vaccination policies based on the priorities. Considering all these facts, a comprehensive but simple parametric model enriched with the pharmacological and non-pharmacological policies has been proposed in this study to analyse and predict the future pandemic casualties.

**Method:**

This paper develops a priority and age specific vaccination policy and modifies the non-pharmacological policies including the curfews, lockdowns, and restrictions. These policies are incorporated with the susceptible, suspicious, infected, hospitalized, intensive care, intubated, recovered, and death sub-models. The resulting model is parameterizable by the available data where a recursive least squares algorithm with the inequality constraints optimizes the unknown parameters. The inequality constraints ensure that the structural requirements are satisfied and the parameter weights are distributed proportionally.

**Results:**

The results exhibit a distinctive third peak in the casualties occurring in 40 days and confirm that the intensive care, intubated, and death casualties converge to zero faster than the susceptible, suspicious, and infected casualties with the priority and age specific vaccination policy. The model also estimates that removing the curfews on the weekends and holidays cause more casualties than lifting the restrictions on the people with the chronic diseases and age over 65.

**Conclusion:**

Sophisticated parametric models equipped with the pharmacological and non-pharmacological policies can predict the future pandemic casualties for various cases.

## Background

The human history has witnessed a number of devastating pandemics such as the smallpox, cholera, plague, dengue, influenzas, Ebola, severe acute respiratory syndrome (SARS), Middle East respiratory syndrome (MERS), and novel coronavirus diseases 2019 (COVID-19) [1]. Since the pandemic diseases mostly spread through human-to-human infection, the non-pharmacological policies including the restrictions, closures, and curfews have been imposed until an effective vaccine has been developed [2]. Even though a vaccine has been developed, problems in its production and distribution create some constraints on fighting the pandemic diseases. In this case, the state authorities seek policies that optimize the respective priorities such as reducing the deaths, easing the curfews, lifting the restrictions, and opening of the schools. This paper proposes a comprehensive parametric model with the priority and age specific vaccination policy which can be used for the prediction and analysis of the future casualties under the constructed policy. In this paper, the healthcare staff constitutes the highest priority group, and the elderly people are located into the risk groups based on their ages.

Accurate models can be a useful tool to understand the dynamics of the pandemic diseases and to identify the role of the internal (mutation) and external (pharmacological and non-pharmacological policies) impacts on the pandemic casualties [3]. Modelling of the pandemic diseases can be achieved by using the non-parametric (statistical and machine learning), and the parametric (mathematical) approaches. Statistical approaches usually reveal a mean and a standard deviation which can be used for characterizing the pandemic properties such as the incubation period and the infectious rate of the pandemic diseases. Overton et al. produced data for the incubation period of the COVID-19 by assuming that it has the Gama distribution and fitted the data with the maximum likelihood estimator [4]. This research stated that the majority of the infected people develop symptoms in 14 days. Hong and Li estimated a time-dependent reproduction number of disease with the Poisson model having a removal rate to account for the random uncertainties in the reported casualties [5]. It is concluded that China, Italy, Sweden, and the United States of America (USA) have high COVID-19 reproduction numbers since they were unable to control the spread of the virus. Oehmke et al. determined the speed, acceleration, jerk, and 7-day-lag in the COVID-19 transmission for the USA and determined the parameters with the Arellano-Bond statistical estimator [6]. It is expressed that there were significant differences in the spread of the virus among the states of the USA due to lack of a national non-pharmacological policy.

In terms of the machine learning based modelling approaches, Pinter et al. proposed an adaptive network-based fuzzy inference systems (ANFIS) and a multi-layered perceptron-imperialist competitive algorithm (MLP-ICA) to estimate the infected individuals and the mortality rate [7]. Tuli et al. considered the generalized Inverse Weibull distribution combined with the cloud computing to predict the growth of the epidemic and to design control strategies for the COVID-19 spread [8]. Aydin and Yurdakul evaluated the policies of the 142 countries against fighting the COVID-19 by using the k-means clustering, decision trees, and random forest algorithms [9]. The research revealed that the economic welfare, smoking rates, and the diabetes rates are not directly related to the effectiveness level of the countries. Rustem et al. utilized the linear regression (LR), the least absolute shrinkage and selection operator (LASSO), the support vector machines (SVM), and the exponential smoothing (ES) to estimate the threating factors of the COVID-19 [10]. The results confirmed that the ES outperforms the others while the SVM performs poorly. Bird et al. evaluated the country-level pandemic risks and classified the preparedness of the countries in terms of the transmission, the mortality, and the inability to test by applying the stack of gradient boosting, the decision trees, the stack of SVM, and the extra trees [11]. It is concluded that the geopolitics and the demographic attributes shape the risks caused by the COVID-19.

Parametric modelling approaches suit their purpose and are also parameterizable by the available data, since they have a certain model structure representing the mathematical relationships as simple as possible [12]. Goel and Sharma proposed a mobility-based susceptible, infected, recovered (SIR) model covering the population distribution and the lockdowns [13]. It is observed that the infected casualties are delayed and decreased in the presence of the lockdowns. Piovella provided a simplified analytical solution of the susceptible, exposed, infected, recovered (SEIR) model to predict the casualty peaks and asymptotic cases without iteratively solving the ordinary differential equations [14]. Even though the numeric and analytical solutions are close, there exist biases around the peak values. Piccolomini and Zama proposed a forced susceptible, exposed, infected, recovered, dead (fSEIRD) model with two different piecewise time-dependent infection rates [15]. It is stated that the model fits the data and makes reliable predictions for Italy. However, even though the SIR, SEIR, fSEIRD models are simple and require few parameters, they do not consider the pharmacological and non-pharmacological policies which play important roles on the dynamics of the pandemic diseases. In addition, they do not include the hospitalized, intensive care, and intubated pandemic casualties. Lee et al. modelled an optimal age specific vaccination policy against the H1N1 pandemic influenza in Mexico [16]. The model suggested that the optimal vaccination can be achieved by allocating more vaccines for the young adults age between 20 and 39. Recently, we developed a suspicious, infected, recovered (SpID) model with the second order difference equations rather than the first order ordinary differential equations as in the SIR, the SEIR, and the SEIRD models [17]. The results confirmed that the SpID model can represent the higher order properties such as the peak in the COVID-19 casualties. In our further research, we proposed a SpID-N model with the non-pharmacological policies (N) including the curfews, restrictions, and lockdowns [2]. The results highlighted the role of each non-pharmacological policy on the COVID-19 casualties. In addition, recently we performed a research to analyses the linear and non-linear dynamics of the COVID-19 by only considering the pharmacological policies [18]. This paper developed three model structures from linear to strongly non-linear and optimized their parameters with the mathematical optimization and machine learning approaches. As an alternative to the model-based control of the pandemic casualties, an artificial intelligence approach, which is implicitly a model free approach, was constructed to generate the multi-dimensional non-pharmacological policies [19]. This artificial intelligence algorithm allowed to weight each non-pharmacological policy together with each pandemic casualty under a certain vaccination policy. It firstly aimed to stabilize the pandemic casualties and then minimize them in time. Zhao et al. recently built an age-specific transmission model to quantify the transmissibility in different age groups [20]. Matrajt et al. developed an optimal vaccine allocation algorithm aiming at reducing the deaths, infections, and hospitalizations [21].

This paper proposes a susceptible (S^c^), suspicious (S^p^), infected (I^n^), hospitalized (H), intensive care (I^t^), intubated (I^b)^, recovered (R), death (D) with the priority and age specific vaccination (V) and non-pharmacological (N) policies (S^c^S^p^I^n^HI^t^I^b^RD-VN model). The key contributions of the paper can be summarized briefly as

**Fig. 1:**
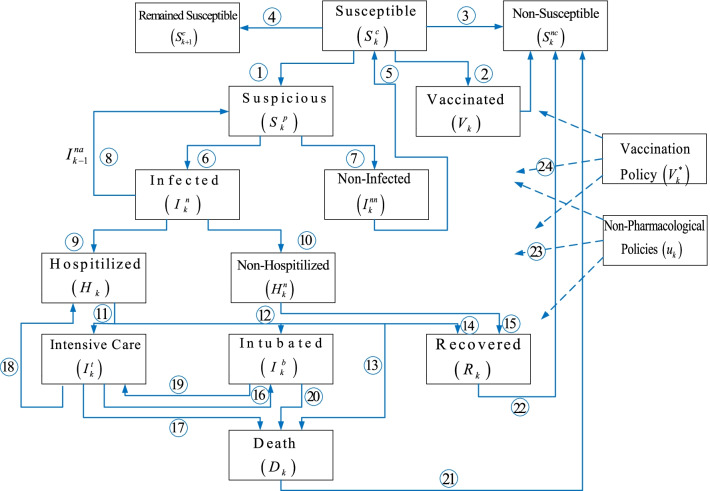
The proposed S^c^S^p^I^n^HI^t^I^b^RD-VN model architecture

- A comprehensive S^c^S^p^I^n^HI^t^I^b^RD-VN model has been constructed by referring the known relationships among the COVID-19 casualties illustrated in Fig. 1.
- Priority and age specific vaccination policy has been formulated and incorporated into the S^c^S^p^I^n^HI^t^I^b^RD-VN model together with the non-pharmacological policies.
- Constrained recursive least squares (RLS) optimizer has been modified to learn the unknown parameters of the S^c^S^p^I^n^HI^t^I^b^RD-VN model by satisfying the structural and proportional contribution requirements of the design.
- An extensive analysis has been performed to assess the role of the priority and age specific vaccination policy and the non-pharmacological policies.

It is important to note that even though this paper mostly refers the COVID-19 pandemic, it can be implemented to all the pandemic diseases having the architecture shown in Fig. 1, which is constructed based on the epidemiological facts. In the rest of the paper, the proposed model structures, the proposed S^c^S^p^I^n^HI^t^I^b^RD-VN model, the constrained RLS for the multi-dimensional models, and the analysis of the model have been provided.

## Methods

### The proposed model architecture

Individuals in the susceptible $\left( {S_{k}^{c} } \right)$ group are vulnerable to the pandemic diseases where the suspicious $\left( {S_{k}^{p} } \right)$ ones leave the group (Fig. 1, number 1) and the non-infected $\left( {I_{k}^{nn} } \right)$ ones re-join the susceptible group (number 5). The vaccinated $\left( {V_{k} } \right)$(number 2), the recovered $\left( {R_{k} } \right)$ (number 22), and the death $\left( {D_{k} } \right)$(number 21) become non-susceptible $\left( {S_{k}^{nc} } \right)$(number 3) and leave the susceptible $\left( {S_{k}^{c} } \right)$ group where the remained ones constitute the current susceptible $\left( {S_{k + 1}^{c} } \right)$(number 4) group. The individuals in the suspicious $\left( {S_{k}^{p} } \right)$ group, who are tested and/or quarantined, either move to the infected $\left( {I_{k}^{n} } \right)$(number 6) group or the non-infected $\left( {I_{k}^{nn} } \right)$(number 7) group where some individuals in the infected $\left( {I_{k}^{n} } \right)$ group can return the suspicious $\left( {S_{k}^{p} } \right)$ group again. Also, since the infected $\left( {I_{k}^{n} } \right)$ individuals spread the virus until they are isolated, they act like as an excitation signal $\left( {I_{k - 1}^{na} } \right)$ (number 8) on the suspicious casualties. Individuals in the infected $\left( {I_{k}^{n} } \right)$ group can be in the hospitalized $\left( {H_{k} } \right)$(number 9) group or in the non-hospitalized $\left( {H_{k}^{n} } \right)$(number 10) group where the non-hospitalized $\left( {H_{k} } \right)$ individuals join the recovered $\left( {R_{k} } \right)$(number 15) group after a quarantine period. The individuals in the hospitalized $\left( {H_{k} } \right)$ group can union with the intensive care $\left( {I_{k}^{t} } \right)$(number 11), the intubated $\left( {I_{k}^{b} } \right)$(number 12), the death $\left( {D_{k} } \right)$(number 13), or the recovered $\left( {R_{k} } \right)$(number 14) groups. The individuals in the intensive care $\left( {I_{k}^{t} } \right)$ group can move to the intubated $\left( {I_{k}^{b} } \right)$(number 16), the death $\left( {D_{k} } \right)$(number 17), or the hospitalized $\left( {H_{k} } \right)$(number 18) groups. Similarly, the individuals in the intubated $\left( {I_{k}^{b} } \right)$ group can join either the intensive care $\left( {I_{k}^{t} } \right)$(number 19), or the death $\left( {D_{k} } \right)$(number 20) groups. The non-pharmacological policies $\left( {u_{k} } \right)$(number 23) and priority and age specific vaccination policy $\left( {V_{k}^{*} } \right)$(number 24) act like an external inhibitor on all the casualties at varying rates.

### The ScSpInHItIbRD-VN model

This section initially formulates the parametric sub-models, and then the vaccination and the non-pharmacological policies of the S^c^S^p^I^n^HI^t^I^b^RD-VN model.

### The S^c^S^p^I^n^HI^t^I^b^RD-VN sub-models

This sub-section constructs the parametric models of each sub-model illustrated in Fig. 1.

### The susceptible $S_{k}^{c}$ sub-model

**Fig. 2:**
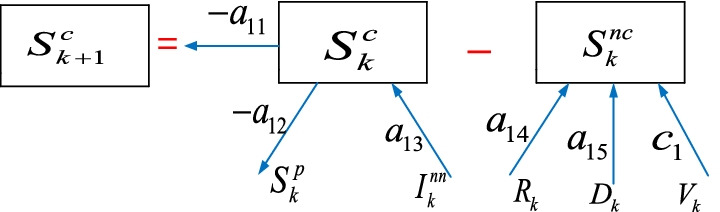
The susceptible $S_{k}^{c}$ sub-model

Considering the connections coming in and leaving out the susceptible $S_{k}^{c}$ group in Fig. 1, one can formulate the $S_{k}^{c}$ sub-model with a difference equation. We can initially write the difference equation of the non-susceptible $S_{k}^{nc}$ group shown in Fig. 2 as

1 $$S_{k}^{nc} = a_{14} R_{k} + a_{15} D_{k} + c_{1} V_{k}$$

where $S_{k}^{nc}$ represents the non-susceptible individuals who have gained immunity and also the individuals who lost their lives, $R_{k}$ represents the recovered individuals, $D_{k}$ represents the dead individuals, $V_{k}$ represents the vaccinated individuals, $a_{14}$,$a_{15}$,$c_{1}$ are the unknown parameters.

The representation of the susceptible $S_{k + 1}^{c}$ group in Fig. 2 is

2 $$S_{k + 1}^{c} = - a_{11} S_{k}^{c} - a_{12} S_{k}^{p} + a_{13} I_{k}^{nn} - S_{k}^{nc}$$

where $S_{k}^{c}$ represents the individuals who may be infected and have a lack of immunity, $S_{k}^{p}$ represents the suspicious individuals, $I_{k}^{nn}$ represents the non-infected individuals, $a_{11}$,$a_{12}$,$a_{13}$ are the unknown parameters.Substituting Eq. (1) in Eq. (2) yields

3 $$S_{k + 1}^{c} = - a_{11} S_{k}^{c} - a_{12} S_{k}^{p} + a_{13} I_{k}^{nn} - a_{14} R_{k} - a_{15} D_{k} - c_{1} V_{k}$$

All the parameters in Eq. (3) are unknown and will be learned from the available data with the RLS algorithm subject to the inequality constraints in the next section.

The next sub-section provides the modelling steps of the suspicious $S_{k}^{p}$ sub-model.

### The suspicious $S_{k}^{p}$ sub-model

**Fig. 3:**
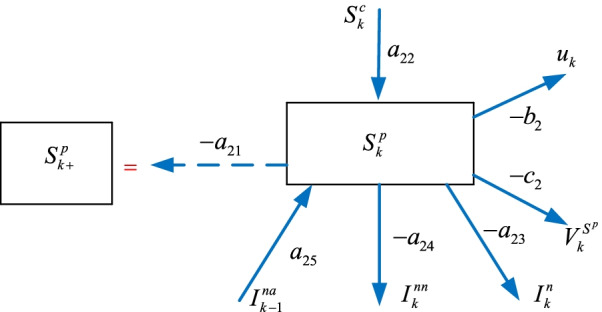
The suspicious $S_{k}^{p}$ sub-model

Some of the susceptible $S_{k}^{c}$ individuals become suspicious $S_{k}^{p}$ as they exhibit symptoms or contact an infected individual, or return from the regions where the pandemic disease is a threat. These individuals are either tested or quarantined for a time duration. In this paper, we define the suspicious $S_{k}^{p}$ individuals as the number of the people tested daily. Therefore, the model can predict the number of the required tests in the future. We can represent the $S_{k}^{p}$ sub-model shown in Fig. 3 as

4 $$\begin{aligned} S_{k + 1}^{p} & = - a_{21} S_{k}^{p} + a_{22} S_{k}^{c} - a_{23} I_{k}^{n} - a_{24} I_{k}^{nn} + a_{25} I_{k - 1}^{na} \ldots \\ & \quad {{ \ldots }} - b_{2} u_{k}^{{}} - c_{2} V_{k}^{{S^{p} }} \\ \end{aligned}$$

where $I_{k}^{na}$ represents the individuals who can become suspicious again and excitation effect of the infected individuals on the suspicious casualties (related to filiation time), $u_{k}^{{}}$ is the non-pharmacological policy, $V_{k}^{{S^{p} }}$ is the vaccination policy, $a_{21}$, $a_{22}$, $a_{23}$, $a_{24}$, $a_{25}$ $b_{2}$, $c_{2}$ are the parameters.

The next sub-section presents the modelling steps of the infected $I_{k}^{n}$ sub-model.

### The infected $I_{k}^{n}$ sub-model

**Fig. 4:**
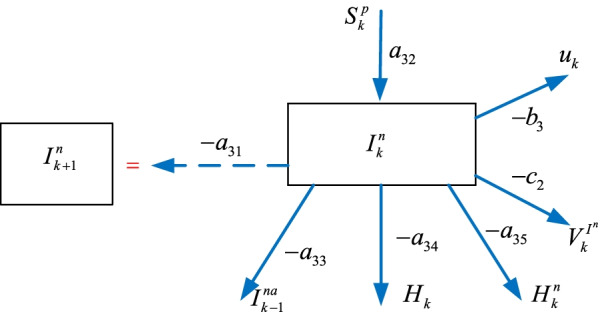
The infected $I_{k}^{n}$ sub-model

Some of the suspicious $S_{k}^{p}$ individuals becomes infected $I_{k}^{n}$ where they either become hospitalized $H_{k}$ or non-hospitalized $H_{k}^{n}$, who are quarantined for a period of time, as illustrated in Fig. 1. We can formulate its model by considering the corresponding connections in Fig. 4 as

5 $$I_{k + 1}^{n} = - a_{31} I_{k}^{n} + a_{32} S_{k}^{p} - a_{33} I_{k - 1}^{na} - a_{34} H_{k}^{{}} - a_{35} H_{k}^{n} - b_{3} u_{k} - c_{3} V_{k}^{{I^{n} }}$$

where $V_{k}^{{I^{n} }}$ is the vaccination policy, $a_{31}$, $a_{32}$, $a_{33}$, $a_{34}$, $a_{35}$ $b_{3}$, $c_{3}$ are the parameters.

The next sub-section introduces the hospitalized $H_{k}$ sub-model.

### The hospitalized $H_{k}$ sub-model

**Fig. 5:**
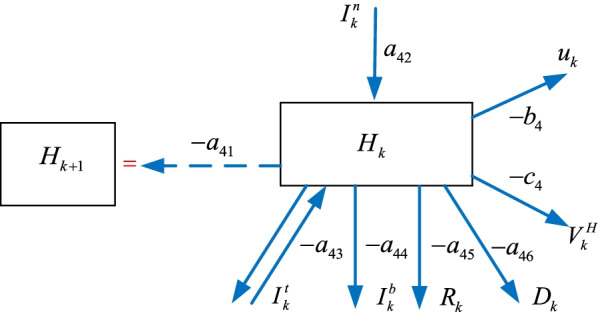
The hospitalized $H_{k}$ sub-model

Some of the infected $I_{k}^{n}$ individuals requiring standard treatments join the hospitalized $H_{k}$ group. The hospitalized $H_{k}$ individuals can join the intensive care $I_{k}^{t}$, the intubated $I_{k}^{b}$, the recovered $R_{k}$, or the death $D_{k}$ groups as shown in Fig. 5. We can formulate the hospitalized model as

6 $$\begin{aligned} H_{k + 1} & = - a_{41} H_{k} + a_{42} I_{k}^{n} - a_{43} I_{k}^{t} - a_{44} I_{k}^{b} - a_{45} R_{k} - a_{46} D_{k} \ldots \\ & \quad \ldots - b_{4} u_{k} - c_{4} V_{k}^{H} \\ \end{aligned}$$

where $V_{k}^{H}$ is the vaccination policy, $a_{41}$, $a_{42}$, $a_{43}$, $a_{44}$, $a_{45}$, $a_{46}$, $b_{4}$, $c_{4}$ are the parameters,

The next sub-section presents the formulation of the intensive care $I_{k}^{t}$ sub-model.

### The intensive care $I_{k}^{t}$ sub-model

**Fig. 6:**
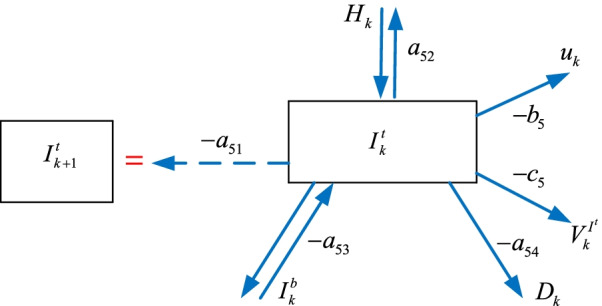
The intensive care $I_{k}^{t}$ sub-model

Some of the hospitalized $H_{k}$ individuals move to the intensive care $I_{k}^{t}$ group where some of them move back to the hospitalized $H_{k}$ group as shown in Fig. 6. Similarly, some of the intensive care $I_{k}^{t}$ patients become intubated $I_{k}^{b}$ where some of them re-join the intensive care $I_{k}^{t}$ group, and the rest join the death $D_{k}$ group. We can construct the intensive care $I_{k}^{t}$ model as

7 $$I_{k + 1}^{t} = - a_{51} I_{k}^{t} + a_{52} H_{k} - a_{53} I_{k}^{b} - a_{54} D_{k} - b_{5} u_{k} - c_{5} V_{k}^{{I^{t} }}$$

where $V_{k}^{{I^{t} }}$ is the vaccination policy, $a_{51}$, $a_{52}$, $a_{53}$, $b_{5}$, $c_{5}$ are the parameters.

The next sub-section provides the intubated $I_{k}^{b}$ sub-model.

### The intubated $I_{k}^{b}$ sub-model

**Fig. 7:**
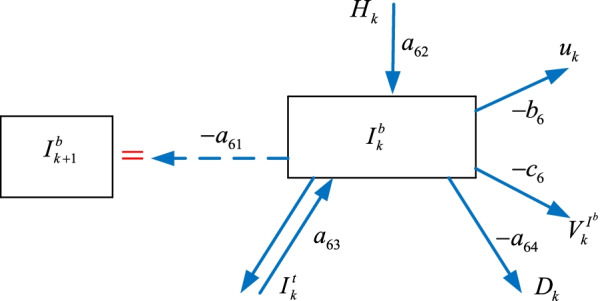
The intubated $I_{k}^{b}$ sub-model

Some of the hospitalized $H_{k}$ individuals and the intensive care $I_{k}^{t}$ patients become intubated $I_{k}^{b}$ as shown in Fig. 7. A number of the intubated $I_{k}^{b}$ patients move back to the intensive care $I_{k}^{t}$ unit while the rest join the death $D_{k}$ group. We can construct the intubated model as

8 $$I_{k + 1}^{b} = - a_{61} I_{k}^{b} + a_{62} H_{k} + a_{63} I_{k}^{t} - a_{64} D_{k} - b_{6} u_{k} - c_{6} V_{k}^{{I^{b} }}$$

where $V_{k}^{{I^{b} }}$ is the vaccination policy, $a_{61}$, $a_{62}$, $a_{63}$, $a_{64}$ $b_{6}$, $c_{6}$ are the parameters.

The next sub-section formulates the recovered $R_{k}$ sub-model.

### The recovered $R_{k}$ sub-model

**Fig. 8:**
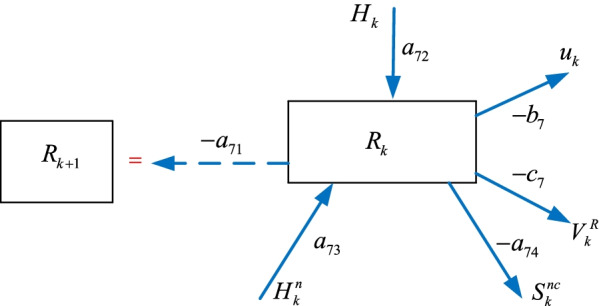
The recovered $R_{k}$ sub-model

A number of the hospitalized $H_{k}$ and the non-hospitalized $H_{k}^{n}$ individuals join the recovered $R_{k}$ group who become non-susceptible $S_{k}^{nc}$ as illustrated in Fig. 8. We can formulate the recovered $R_{k}$ sub-model as

9 $$\begin{aligned} R_{k + 1} & = - a_{71} R_{k} + a_{72} H_{k} + a_{73} H_{k}^{n} - a_{74} S_{k}^{nc} \ldots \\ & \quad \ldots - b_{7} u_{k} - c_{7} V_{k}^{R} \\ \end{aligned}$$

where $V_{k}^{R}$ is the vaccination policy, $a_{71}$, $a_{72}$, $a_{73}$, $a_{74}$ $b_{7}$, $c_{7}$ are the parameters.

The next sub-section expresses the death sub-model.

### The death $D_{k}$ sub-model

**Fig. 9:**
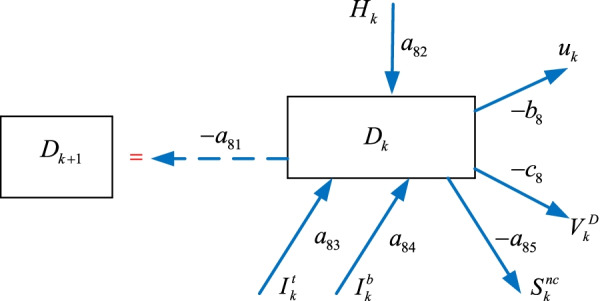
The death $D_{k}$ sub-model

Some of the hospitalized $H_{k}$, the intensive care $I_{k}^{t}$, and the intubated $I_{k}^{b}$ individuals join the death $D_{k}$ group and become non-susceptible $S_{k}^{nc}$ as illustrated in Fig. 9. We can form the death $D_{k}$ model as

10 $$\begin{aligned} D_{k + 1} & = - a_{81} D_{k} + a_{82} H_{k} + a_{83} I_{k}^{t} + a_{84} I_{k}^{b} - a_{85} S_{k}^{nc} \ldots \\ & \quad \ldots - b_{8} u_{k} - c_{8} V_{k}^{D} \\ \end{aligned}$$

where $V_{k}^{D}$ is the vaccination policy, $a_{81}$, $a_{82}$, $a_{83}$, $a_{84}$, $a_{85}$ $b_{8}$, $c_{8}$ are the parameters.

The next sub-section formulates the vaccination policy $V_{k}^{*}$ and reviews the non-pharmacological $u_{k}$ policies.

### The vaccination $V_{k}^{*}$ and non-pharmacological $u_{k}$ policies

This section firstly introduces the priority and age specific vaccination policy $V_{k}^{*}$ and reviews the non-pharmacological policies $u_{k}$ that we have developed recently for the first time in the literature [2].

### The priority and age specific vaccination policies $V_{k}^{*}$

The $*$ in the priority and age specific vaccination policy $V_{k}^{*}$ represents the $S^{c}$,$S^{p}$,$I^{n}$,$H$,$I^{t}$,$R$, and $D$ in the sub-models given by Eqs. from (4) to (10). The priority and age specific vaccination policy basis $V_{k}^{b}$ is defined in terms of the number of the daily vaccinated people in each group as

11 $$V_{k}^{b} = \left[ {\begin{array}{*{20}c} {H_{k}^{s} } & {A_{{^{k} }}^{80 + } } & {A_{k}^{65 - 79} } & {A_{k}^{50 - 64} } & {A_{k}^{25 - 49} } & {A_{k}^{15 - 24} } \\ \end{array} } \right]^{T}$$

where $H_{k}^{s}$ is the healthcare staff, $A_{k}^{80 + }$ is the people age 80 and over, $A_{k}^{65 - 79}$ is the people age between 65 and 79, $A_{k}^{50 - 64}$ is the people age between 50 and 64, $A_{k}^{25 - 49}$ is the people age between 25 and 49, $A_{k}^{15 - 24}$ is the people age between 15 and 24.

Since the people age under 15 are not considered for the vaccination, they are not included in the basis $V_{k}^{b}$. The corresponding weight parameter vector $w_{k}^{*}$ scales the contribution of the vaccination policy for each sub-model. For example, the weight parameter vector for the hospitalized $w_{k}^{H}$ is

12 $$w_{k}^{H} = \left[ {\begin{array}{*{20}c} {w_{k}^{s} } & {w_{k}^{80 + } } & {w_{k}^{65 - 79} } & {w_{k}^{50 - 64} } & {w_{k}^{25 - 49} } & {w_{k}^{15 - 24} } \\ \end{array} } \right]^{T}$$

where the parameters of the $w_{k}^{H}$ are $w_{k}^{s}$ is the percentage of the hospitalized $H_{k}^{s}$, $w_{k}^{80 + }$ is the percentage of the hospitalized $A_{k}^{80 + }$, $w_{k}^{65 - 79}$ is the percentage of the hospitalized $A_{k}^{65 - 79}$, $w_{k}^{50 - 64}$ is the percentage of the hospitalized $A_{k}^{50 - 64}$, $w_{k}^{25 - 49}$ is the percentage of the hospitalized $A_{k}^{25 - 49}$, $w_{k}^{15 - 24}$ is the percentage of the hospitalized $A_{k}^{15 - 24}$.

Now we can formulate the priority and age specific vaccination policy for the hospitalized $V_{k}^{H}$ in Eq. (6) as

13 $$V_{k}^{H} = w_{k}^{{H}{^{T} }} V_{k}^{b}$$

Similarly, we can construct the priority and age specific vaccination policy $V_{k}^{*}$ for the other sub-models by following the same steps introduced in this section. The next sub-section provides the revised non-pharmacological policies $u_{k}$.

### The non-pharmacological policies $u_{k}$

The authorities impose various curfews and restrictions to confine the spread of the virus. The most common ones are the curfews on the people age over 65, age under 20, and people with the chronic diseases which have been parametrized in [2] (since there is no available data) as

14 $$u_{k}^{s} = n^{s} \left( {1 - \alpha^{{k - k_{i} }} + \sigma_{k}^{s} } \right)$$

where $u_{k}^{s}$ is the response of the curfew (in closed form solution), $n^{s}$ is the number of the people under the curfew, $k$ is the number of the days and $k_{i}$ is the start day of the curfew, $\alpha$ is the discount factor of the response, where $\alpha^{k} \approx 0$ for $\alpha = 0.71$ and $k = 14$ (quarantine duration), $\sigma_{k}^{s}$ is the random non-parametric uncertainty in the response.

The other common precaution is the curfews on the weekends and holidays, which has a transient ascent part as

15 $$\begin{aligned} u_{i,k}^{wh} & = n^{wh} \left( {1 - \alpha^{k - i} + \sigma_{i,k}^{wh} } \right)\delta_{i,k} \ldots \\ & \quad \quad ...{\text{for }}\left\{ {\begin{array}{*{20}l} {\delta_{i,k} = 1} \hfill & {\left\{ {\begin{array}{*{20}c} {{\text{Curfew}}\,{\text{at}}\,ith\,{\text{day}}} \\ {i \le k \le i + 6} \\ \end{array} } \right.} \hfill \\ {\delta_{i,k} = 0} \hfill & {{\text{Otherwise}}} \hfill \\ \end{array} } \right. \\ \end{aligned}$$

where $u_{i,k}^{wh}$ is the response of the curfews on the weekends and holidays, $n^{wh}$ is the number of the people under the curfews on the weekends and holidays, $\sigma_{i,k}^{wh}$ is the random uncertainty in the response.

Its transient descent part is modelled as

16 $$\begin{aligned} u_{i,k}^{wh} & = n^{wh} \left( {\alpha^{k - i} + \sigma_{i,k}^{wh} } \right)\delta_{i,k} \ldots \\ & \quad \quad \ldots {\text{for}}\left\{ {\begin{array}{*{20}l} {\delta_{i,k} = 1} \hfill & {\left\{ {\begin{array}{*{20}c} {{\text{Curfew}}\,{\text{at}}\,ith\,{\text{day}}} \\ {i + 7 \le k \le i + 14} \\ \end{array} } \right.} \hfill \\ {\delta_{i,k} = 0} \hfill & {{\text{Otherwise}}} \hfill \\ \end{array} } \right. \\ \end{aligned}$$

The overall response $u_{k}^{wh}$ is

17 $$u_{k}^{wh} = \sum\limits_{i = k - 14}^{k} {u_{i,k}^{wh} }$$

In terms of the closure of the schools and universities, it is not a curfew as it only hinders mass gatherings of the students; hence, they can come together in smaller groups. Therefore, the response has a transient ascent part as in Eq. (15) and transient descent part as in Eq. (16). These parts are essentially for removing the negative impacts of the schools being open. Then an uncertain saturated part $u_{sat}$ represents the small gatherings after the closure of the schools. After the transient ascent and descent parts, the saturated part can be represented as

18 $$u_{k}^{su} = n^{su} \left( {u_{sat} + \sigma_{k}^{su} } \right)\quad {\text{for}}\quad k = k_{i}^{su} , \ldots ,k_{n}$$

where $n^{su}$ is the number of the students, $\sigma_{k}^{su}$ is the random uncertainty in the response, $k_{n} = k_{i}^{su} + k_{n}^{su}$ where $k_{i}^{su}$ is the start day and $k_{n}^{su}$ is the duration of the closure.

### Comparison of the prediction models

One can summarize the main advantages of the constructed S^c^S^p^I^n^HI^t^I^b^RD-VN model over the well-known models such as the SIR, SEIR models in terms of the solution and analysis as

- It has difference equations rather than the differential equations. Therefore, it can be solved iteratively without requiring an ordinary differential equation solver.
- It has coupled and linear dynamics instead of the slightly coupled nonlinear dynamics. Thus, the mathematical analysis of the parametric model is straightforward.
- Its unknown parameters are learned from the reported data by using the well-known multi-dimensional optimization approaches rather than the single dimensional statistical approaches.

The next section forms the RLS approach with the inequality constraints to learn the unknown parameters of the S^c^S^p^I^n^HI^t^I^b^RD-VN model.

### The constrained RLS algorithm

In this paper, the constrained optimization is considered for two reasons: The first one is that the sub-models have certain parameter structures together with the corresponding parameter signs and the second reason is to reflect the contributions of the data having huge magnitude differences (for example, while the susceptible $S_{k}^{c}$ group covers millions of the individuals, the hospitalized $H_{k}$ group covers only thousands of them). In this section, initially we will divide the optimization problem in terms of the estimated sub-model casualties (outputs) and the real casualties. Then, the RLS algorithm with the inequality constraints are modified to learn the unknown parameters.

### The estimated sub-models

We can represent the estimated sub-models $\hat{y}_{k}^{*}$ in terms of the known basis vector $b_{k}^{*}$ and the unknown parameter vector $w_{k}^{*}$, where the $*$ is denoted for the $S^{c}$,$S^{p}$,$I^{n}$,$H$,$I^{t}$,$R$, and $D$ in the sub-models given by Eqs. from (3) to (10) as

19 $$\hat{y}_{k}^{*} = w_{k}^{{*^{T} }} b_{k}^{*}$$

For example, the basis $b_{k}^{{S^{c} }}$ of the estimated susceptible $\hat{y}_{k}^{{S^{c} }}$ sub-model is formed with respect to the left hand side of Eq. (3) as

20 $$b_{k}^{{S^{c} }} = \left[ {\begin{array}{*{20}c} { - S_{k}^{c} } & { - S_{k}^{p} } & {I_{k}^{nn} } & { - R_{k} } & { - D_{k} } & { - V_{k} } \\ \end{array} } \right]^{T}$$

And the corresponding unknown parameter vector $w_{k}^{{S^{c} }}$ of the estimated susceptible $\hat{y}_{k}^{{S^{c} }}$ sub-model with respect to the right hand side of Eq. (3) is

21 $$w_{k}^{{S^{p} }} = \left[ {\begin{array}{*{20}c} {a_{11} } & {a_{12} } & {a_{13} } & {a_{14} } & {a_{15} } & {c_{1} } \\ \end{array} } \right]$$

The other estimated sub-models, their bases and parameter vectors are formed by following the same procedures as in Eqs. (19), (20), and (21), respectively. The next sub-section introduces the modified RLS algorithm with the inequality constraints to learn the unknown parameter vectors $w_{k}^{*}$.

### Learning the Unknown Parameters with the Constrained RLS

The reported casualties are the outputs of the S^c^S^p^I^n^HI^t^I^b^RD-VN sub-models and we call them as the real outputs $y_{k}^{*}$. For example, the real output of the susceptible sub-model is the left hand side of Eq. (3), which is $S_{k + 1}^{c}$. The objective function is constructed with the 2-norm of the instant estimation error defined as

22 $$\begin{array}{*{20}l} {\mathop {{\text{min}}}\limits_{{w_{k}^{*} }} } \hfill & {\frac{1}{2}\left\| {y_{k}^{*} - \hat{y}_{k}^{*} } \right\|_{2} } \hfill \\ {{\text{subject}}\,{\text{to}}} \hfill & {\left\| {w_{k}^{*} } \right\|_{2} \le \alpha } \hfill \\ \end{array}$$

where $\alpha$ is the inequality constraints which are the lower bound of the parameters. We can construct the Lagrange multipliers used for solving the optimization problems as

23 $$L\left( {w_{k}^{*} ,\lambda } \right) = \frac{1}{2}\left\| {y_{k}^{*} - w_{k}^{{*^{T} }} b_{k}^{*} } \right\|_{2}^{2} + \frac{\lambda }{2}\left( {\left\| {w_{k}^{*} } \right\|_{2}^{2} - \alpha_{2}^{2} } \right)$$

Getting partial derivative of $L\left( {w_{k}^{*} ,\lambda } \right)$ with respect to the $w_{k}^{*}$ yields

24 $$\left( {b_{k}^{{*^{T} }} b_{k}^{*} + \lambda } \right)w_{k}^{*} = b_{k}^{{*^{T} }} y_{k}^{*}$$

Getting partial derivative of $L\left( {w_{k}^{*} ,\lambda } \right)$ with respect to the $\lambda$ gives

25 $$\left\| {w_{k}^{*} } \right\|_{2}^{2} - \alpha^{2} = 0$$

Re-organizing Eq. (24) as $w_{k}^{*}$ is on the left and the rest are on the right, and then substituting it in Eq. (25) yields

26 $$\left( {\frac{{b_{k}^{*} y_{k}^{*} }}{{b_{k}^{{*^{T} }} b_{k}^{*} + \lambda }}} \right)^{2} - \alpha^{2} = 0$$

The Lagrange multiplier $\lambda$ from Eq. (26) is obtained as

27 $$\lambda = \left( {b_{k}^{{*^{T} }} y_{k}^{*} - \alpha b_{k}^{{*^{T} }} b_{k}^{*} } \right)/\alpha$$

Then by reinserting Eq. (27) into Eq. (24), the unknown parameter vector $w_{k}^{*}$ can be attained. The next section extensively analyses the S^c^S^p^I^n^HI^t^I^b^RD-VN model.

## Results

This section initially presents the parameters of the proposed S^c^S^p^I^n^HI^t^I^b^RD-VN model and then analyses the training and prediction results.

### Parameters of the model

Table 1 provides the parameters of the model.

**Table 1:** Parameters of the models

$A^{80 + }$	1.527.789	Age 80 and over
$A^{65 - 79}$	6.425.766	Age between 65 and 79
$A^{50 - 64}$	12.273.613	Age between 50 and 64
$A^{25 - 49}$	30.962.207	Age between 25 and 49
$A^{15 - 24}$	12.893.753	Age between 15 and 24
$H$	1.061.635	Healthcare staff
$n^{su}$	26.048.00	Student numbers
$n^{wh}$	82.154.00	People under curfew on the weekends

The next sub-section compares the real and estimated COVID-19 casualties with the constrained RLS algorithm.

### Real and estimated casualties

**Fig. 10:**
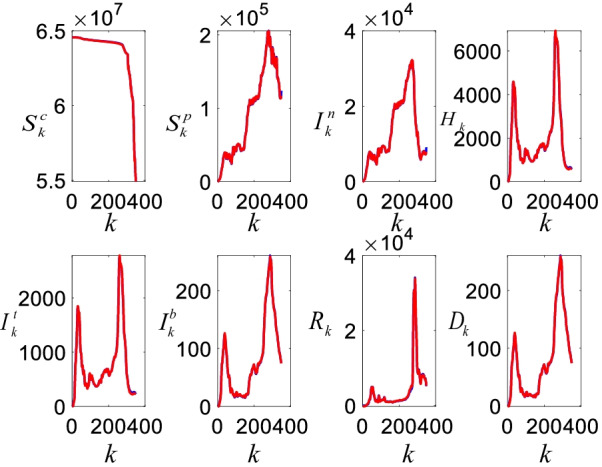
The real (blue) and estimated (red) COVID-19 casualties for Turkey

Figure 10 shows the real (reported) and estimated casualties for Turkey.

**Fig. 11:**
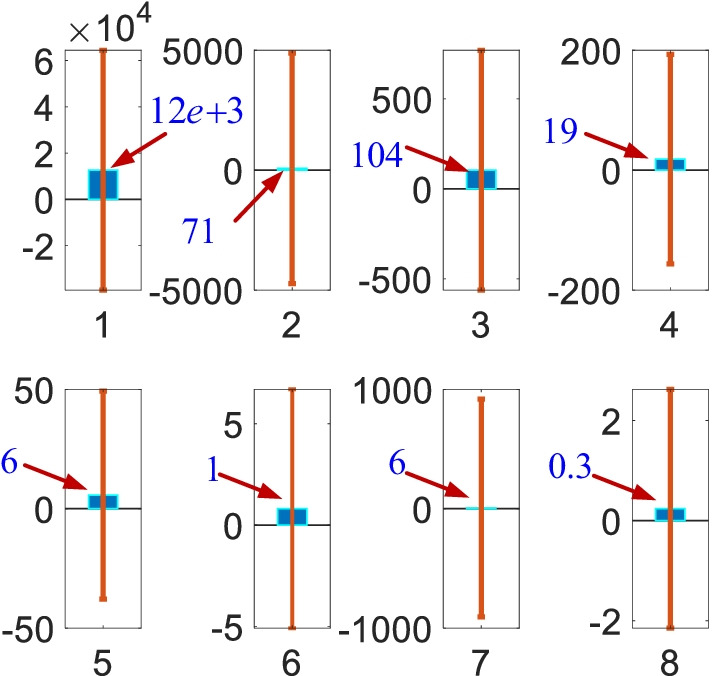
Mean errors (blue bars) and standard deviations (vertical lines). The numbers 1 to 8 represent corresponding estimates with the $S^{c}$, $S^{p}$, $I^{n}$, $H$, $I^{t}$, $R$, and $D$ sub-models, respectively

As can be seen from Fig. 10, the estimated casualties with the S^c^S^p^I^n^HI^t^I^b^RD-VN model closely follow the real casualties. The model can track the steep peaks and also the daily variations in the casualties even though the constructed parameter spaces are limited (in machine learning approaches, we randomly manipulate the parameter spaces until we have close estimations). The casualties in Fig. 10 have two distinctive peaks and estimated future casualties in Fig. 13 shows the third peak, which will occur in 40 days. It is clear that the susceptible $S_{k}^{c}$ casualties have noticeable reduction with the initiation of the vaccination process. It seems that this vaccination process has affected the other casualties since they sharply decrease as well. The decrease in the casualties has also been supported with the non-pharmacological policies $u_{k}$. Figure 11 shows the mean errors and the corresponding standard deviations in the estimates.

As illustrated by Fig. 11, even though all the mean errors are small, the standard deviations are quite large. This is due to existence of the steep peaks shown in Fig. 10. These peaks occurred in December when there were not any active pharmacological and non-pharmacological policies. This implies that the character of the casualties is largely shaped based on the external impacts such as the pharmacological and non-pharmacological policies. Our recent work highlighted that the pharmacological and non-pharmacological policies have damping impact on the casualties whereas they also have natural frequency determined by the internal and coupling dynamics. The next sub-section presents the priority and age specific vaccination policy results.

### Priority and age specific vaccination policy

**Fig. 12:**
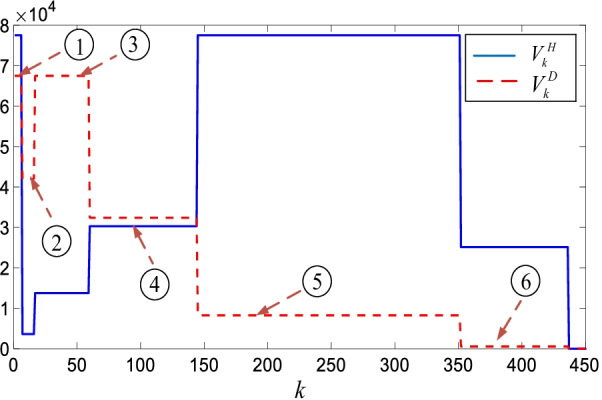
Priority and age specific vaccination policy for the hospitalized $V_{k}^{H}$ and death $V_{k}^{D}$ sub-models

Figure 12 shows the priority and age specification vaccination policy for the hospitalized $V_{k}^{H} = w_{k}^{{H^{T} }} V_{k}^{b}$ and death $V_{k}^{D} = w_{k}^{{D^{T} }} V_{k}^{b}$ sub-models.

Table 2 provides the background parameters in Fig. 12.

**Table 2:** Priority and age specific vaccination parameters

	1	2	3	4	5	6
$w_{k}^{H}$	51	2.4	9.1	20.2	51.7	16.7
$w_{k}^{D}$	45	28	45	21.6	5.5	3.3
$V_{k}^{b}$	1.2	1.8	7.8	14	37	15

Since there is no reported data for the healthcare staff $H^{s}$(‘Staff’ in Table 1), the largest values are assigned for them as they are in the highest risk group. Therefore, the vaccination of the healthcare staff has the largest hospitalized $w_{k}^{H}$ and death $w_{k}^{D}$ policy values (Table 2, number 1). People age 80 and over $\left( {A^{80 + } } \right)$ has 28% death percentage and 2.4% hospitalization percentage. Henceforth, the corresponding $V_{k}^{D}$ is larger than the $V_{k}^{H}$(number 2). Same comments are valid for the people age between 65 and 79 $\left( {A^{65 - 79} } \right)$ (number 3) as they share the similar percentages. However, with respect to the people having age 50 and 64, they have close $w_{k}^{H}$ and $w_{k}^{B}$ parameters; hence, the $V_{k}^{H}$ and $V_{k}^{D}$ policy values are close to each other (number 4). For the people age between 25 and 49 $\left( {A^{25 - 49} } \right)$, $w_{k}^{H}$ is 51.7 and $w_{k}^{D}$ is 5.5; thus, the corresponding $V_{k}^{H}$ is larger than the $V_{k}^{D}$(number 5). Similar comments can be made for the people age between 15 and 24 (number 6). Lastly, since the healthcare staff $H^{s}$ and people age 80 and over $\left( {A^{80 + } } \right)$ have the smallest population among the all age groups, they have the smallest regions on the horizontal axis representing the number of days $k$.

### The estimated future casualties

**Fig. 13:**
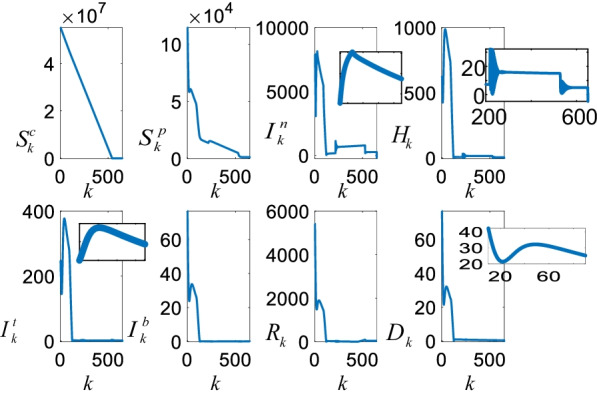
Future COVID-19 estimates with the S^c^S^p^I^n^HI^t^I^b^D-VN model

Figure 13 shows the future casualties estimated by the S^c^S^p^I^n^HI^t^I^b^D-VN model.

The future estimates are obtained under the assumption that 100.000 peoople are vaccinated daily. It is clear that the number of the susceptible $S_{k}^{c}$ individuals reduces almost linearly since the vaccinated $V_{k}$ individuals leave the group. Since the susceptible $S_{k}^{c}$ and the suspicious $S_{k}^{p}$ groups are strongly coupled, reduction in the susceptible $S_{k}^{c}$ group is reflected onto the suspicious $S_{k}^{p}$ group as well. Figure 13 also clearly shows the third peak in the COVID-19 casualties. In addition, it is noticaeble that even though the suspicious $S_{k}^{p}$, the infected $I_{k}^{n}$, and the hospitalized $H_{k}$ converge to zero in 500 days, the intensive care $I_{k}^{t}$, the intubated $I_{k}^{b}$, and the death $D_{k}$ converge to zero around 120 days. This fast convergence is due to priority and age specific vaccination policy which focus on vaccination of the people in the high risk groups.

### Analysis of the vaccination policy

**Fig. 14:**
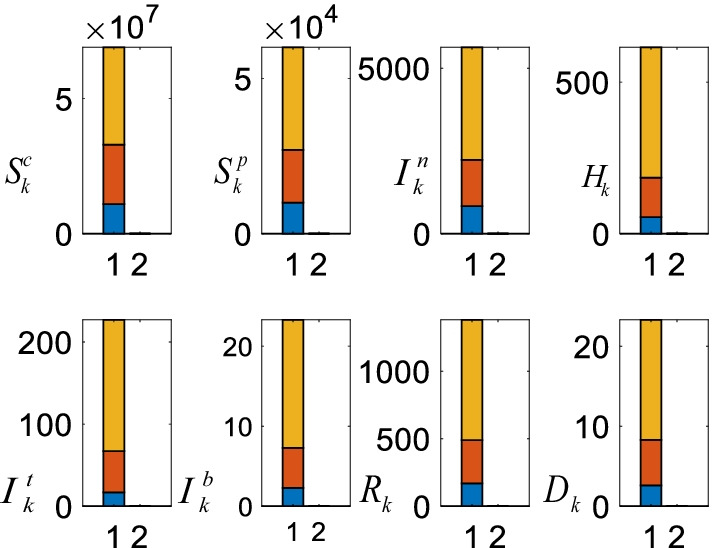
Daily vaccinations and the corresponding casualties. The bottom (blue), the middle (orange), the top (yellow) represent 200.000, 100.000, and 50.000 daily vaccinations, respectively

Figure 14 shows the number of the daily vaccinations and the corresponding average future casualties.

As can be clearly seen from Fig. 14, all the COVID-19 casualties reduce depending on the number of the daily vaccinations. The largest reductions occur in the number of the intensive care $I_{k}^{t}$, the intubated $I_{k}^{b}$, and the death $D_{k}$ when the number of the vaccination rises from 50.000 to 100.000. The further noticeable reduction occurs in the number of the suspicious $S_{k}^{p}$ and the infected $I_{k}^{n}$ when the daily vaccination number rises from 100.000 to 200.000.

#### Analysis of the non-pharmacological policies

**Fig. 15:**
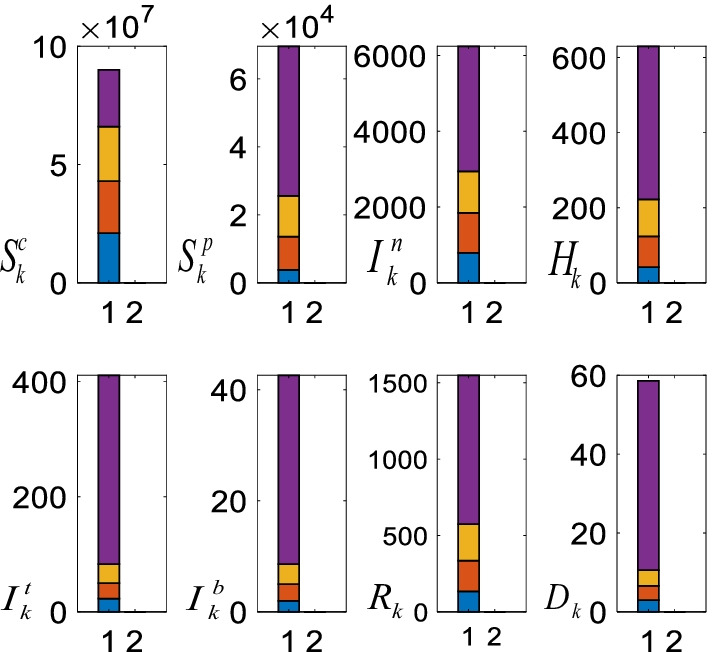
Non-pharmacological policies and the casualties under 100.000 daily vaccination assumption. The average casualties when all the non-pharmacological policies are active (blue), without restrictions on the people age over 65, age under 20, and people with chronic diseases (orange), schools are partially open (yellow), and without the curfews on the weekends and holidays (purple)

Figure 15 shows the role of the non-pharmacological policies on the casualties.

As can be seen from Fig. 15 when all the non-pharmacological policies are in place, all the casualties are small and they increase when the curfews are lifted (blue bar). Removing the restrictions on the people age over 65 and people with the chronic diseases has limited effects as they are in the priority group and most of them have been already vaccinated (orange bar). With respect to the partial opening of the schools, since the majority of the students are not attending the schools, their impact is bounded as well (yellow bar). However, curfews on the weekends, holidays, and nights cover the whole population; henceforth, their roles on the casualties are distinctive (purple bar).

## Conclusions

This paper developed a comprehensive parametric S^p^S^c^I^n^HI^t^I^b^RD-VN model to analyse and estimate the role of the priority and age specific vaccination policy and the non-pharmacological policies. The model has a structure constructed by using the key insights about the pandemic diseases. To satisfy the model structural requirements and avoid dominant effects of the large susceptible and suspicious data, a constrained RLS algorithm has been formed. The results clearly show the importance of the priority and age specific vaccination policy on all the casualties. The future hospitalized, intensive care, intubated, and death casualties converge to zero before the other casualties since they have larger importance. However, the future susceptible, suspicious, infected, and recovered casualties are large due to the people in the lower risk groups are not vaccinated yet. The paper also addresses the relationships among the various daily vaccinations, non-pharmacological polices, and the corresponding COVID-19 casualties. The results confirm that the curfews on the weekends and holidays has an overwhelming role on reducing the casualties.

### Limitations of the work

Effects of the non-pharmacological policies on each age and chronic diseases group are not weighted. Moreover, vaccine effectiveness for each age group has not been added the model. Besides, climate and environmental effects are not considered.

### 4.2 Future works

The non-pharmacological policies of the S^c^S^p^I^n^HI^t^I^b^RD-VN model should be also modified to consider the priority and age specific impacts on each casualty. In addition, effectiveness of the different brand of vaccines on each age group should be considered in the model. Moreover, the model can be expanded by considering the unknown uncertainties. Finally, a toolbox should be constructed and provided for free to help the researchers when applying the proposed model.

## Data Availability

The data are available from: https://corona.cbddo.gov.tr/.

## Abbreviations

SARS: Severe acute respiratory syndrome
MERS: Middle East respiratory syndrome
COVID-19: Novel coronavirus diseases
ANFIS: Adaptive network-based fuzzy inference systems
MLP-ICA: Multi-layered perceptron-imperialist competitive algorithm
LR: Linear regression
LASSO: Least absolute shrinkage and selection operator
SVM: Support vector machines
ES: Exponential smoothing
